# Exosomal hsa-miR-151a-3p and hsa-miR-877-5p are potential novel biomarkers for predicting bone metastasis in lung cancer

**DOI:** 10.18632/aging.205314

**Published:** 2023-12-18

**Authors:** Kun Zhao, Changji Jia, Jin Wang, Weiye Shi, Xiaoying Wang, Yan Song, Changliang Peng

**Affiliations:** 1 Department of Spinal Surgery, The Second Hospital of Shandong University, Jinan 250033, China; 2 Department of Pathology, The Second Hospital of Shandong University, Jinan 250033, China; 3 Department of Nephrology, The Second Hospital of Shandong University, Jinan 250033, China

**Keywords:** lung cancer, bone metastasis, exosome, biomarker, miRNAs

## Abstract

Exosomal miRNAs (exo-miRNAs) have arisen as novel diagnostic biomarkers for various cancers. However, few reports on exo-miRNAs related to bone metastasis (BM) in lung cancer exist. This study aims to screen out key exo-miRNAs and estimate their prognostic values for predicting BM in lung cancer. The differentially expressed exo-miRNAs between the highly-metastatic (95D) and lowly-metastatic (A549) human lung cancer cell lines were comprehensively analyzed using high-throughput sequencing followed by bioinformatic analyses. 29 candidate exo-miRNAs were identified, and 101 BM-related target genes were predicted. Enrichment analysis revealed that these target genes were mainly involved in regulating transcription and pathways in cancer. An exosomal miRNA-mRNA regulatory network consisting of 7 key miRNAs and 10 hub genes was constructed. Further function analysis indicated that these 10 hub genes were mainly enriched in regulating cancer’s apoptosis and central carbon metabolism. The survival analysis indicated that 7 of 10 hub genes were closely related to prognosis. Mutation analysis showed that lung cancer patients presented certain genetic alterations in the 7 real hub genes. GSEA for a single hub gene suggested that 6 of 7 real hub genes had close associations with lung cancer development. Finally, ROC analysis revealed that hsa-miR-151a-3p and hsa-miR-877-5p provided high diagnostic accuracy in discriminating patients with bone metastasis (BM+) from patients without bone metastasis (BM-). These findings provided a comprehensive analysis of exo-miRNAs and target genes in the regulatory network of BM in lung cancer. In particular, hsa-miR-151a-3p and hsa-miR-877-5p may be novel biomarkers for predicting BM in lung cancer.

## INTRODUCTION

Lung cancer is the most common leading cause of cancer-related deaths worldwide [1, 2]. Bone is one of the most common sites of lung cancer metastasis, and the incidence of bone metastasis (BM) in lung cancer is about 30–40% [3]. Once BM occurs, the prognosis for lung cancer patients is generally very poor [4]. The median survival time of patients with BM is only 6–10 months, and the 1-year survival rate after treatment is only 40–50% [5]. Skeletal-related events (SREs) caused by BM, such as bone pain, pathological fractures, spinal cord compression, hypercalcemia, etc., can significantly shorten the survival time of lung cancer patients [6]. Some studies have shown that the survival time can be even shortened by half [4, 6]. Patients with BM have a poor prognosis mostly due to the advanced stage at diagnosis [7]. Usually, imaging diagnostic techniques such as X-ray, computed tomography, magnetic resonance imaging, technetium 99m-methyl diphosphonate (99mTc-MDP) bone scan, and 18F-fluorodeoxyglucose positron emission tomography/computed tomography are used to confirm the diagnosis of a patient with BM [8, 9]. Unfortunately, the low specificity, invasive nature, intricate method, and high cost of these procedures limit their practical applicability. Several studies have demonstrated that bone-specific alkaline phosphatase (BAP), parathyroid hormone-related peptide (PTHrP), type I collagen cross-linked C-telopeptide (ICTP), N-telopeptide (NTx), C-terminal telopeptide (CTx), pyridinoline (PYD), and deoxypridinoline (DPD) may function as sensitive biomarkers for the early detection of BM in lung cancer [10–12]. Nevertheless, these bone metabolic markers can also be detected in patients with BM resulting from breast [13], prostate [14], or kidney cancer [15], and there is currently a lack of specific biomarkers for BM in lung cancer. Additionally, there are numerous types of reagents and detection techniques, and there is a considerable biological variety in bone metabolic markers [16]. To date, a single, global standard does not exist. Therefore, finding new biomarkers for the early diagnosis of BM in lung cancer is crucial to improving the prognosis of lung cancer patients.

MicroRNA (miRNA), as an important mediator in epigenetic control of gene expression, has been increasingly reported to serve as a potential diagnostic and prognostic biomarker as well as a therapeutic target for a variety of cancers [17]. Emerging evidence indicates that miRNAs can regulate diverse biological processes and play critical roles in the development and progression of many kinds of cancer through post-transcriptional regulation mechanisms [18]. Recently, several dysregulated miRNAs are involved in the pathogenesis of lung cancer by affecting cell proliferation, apoptosis, migration, and metastasis [19]. Through genome-wide investigation and analysis, a set of miRNAs were found to be deregulated in the tissues of lung cancer patients with BM, and a group of miRNAs are closely correlated to prognosis and can be used as diagnostic biomarkers [20, 21]. Although the role of miRNAs in regulating BM has been indicated, the underlying molecular mechanism is still poorly understood, especially in lung cancer.

Exosomes, secreted by cells, are vesicles with a diameter of 30–150 nm, composed of lipid bilayer structure, and carry proteins, lipids, DNA, mRNA, and miRNAs [22]. Studies have demonstrated that exosomes play a fundamental role in cells to communicate with neighboring or with distant cells [23, 24]. Due to exosomes’ wide distribution in various human body fluids (blood, urine, saliva, cerebrospinal fluid, ascites, follicular fluid, and joint fluid), exosomes are regarded as ideal non-invasive biomarkers for early disease diagnosis [25].

Exosomal miRNAs (exo-miRNAs) in biofluids are more stable than free miRNAs in circulation [24]. Current studies have shown that exo-miRNAs can be used as diagnostic and prognostic biomarkers in human malignant tumors, including gliomas, ovarian cancer, breast cancer, and so on [26, 27]. Recently, several reports have revealed that certain miRNAs have great potential to be used as diagnostic, predictive, and prognostic biomarkers in BM in lung cancer [28, 29]. However, these studies have limitations, either they did not combine exosomes or they did not combine receiver operating characteristics (ROC) analysis to evaluate the diagnostic value of miRNAs, so their research conclusions are not fully convincing. Moreover, there is a lack of comprehensive and systematic research on the biological role of exo-miRNAs as BM mediators in lung cancer, which warrants further investigations.

In this study, we compared the differentially expressed miRNAs (DE-miRNAs) of highly-metastatic lung cancer cell-derived exosomes and lowly-metastatic lung cancer cell-derived exosomes by high-throughput sequencing. In addition, we used integrated bioinformatics analysis to reveal the miRNA-mRNA interaction network, the potential biological functions, and the key pathways of these common differential miRNAs. We recognized hsa-miR-151a-3p and hsa-miR-877-5p as novel biomarkers for early detection of BM in lung cancer, which helps screen the patients at high risk of BM for early intervention, thereby improving patient outcomes and reducing treatment costs.

## MATERIALS AND METHODS

### Cell lines and cell culture

The highly-metastatic human lung cancer cell line 95D and the lowly-metastatic human lung cancer cell line A549 were purchased from the American Type Culture Collection (ATCC, USA). Both cell lines were cultured in DMEM medium supplemented with 10% exosome-depleted fetal bovine serum (FBS, Gibco, USA), and incubated at 37°C in a humidified atmosphere with 5% CO_2_.

### Exosome isolation

Exosome isolation from the cell culture medium was performed as described previously [30]. Briefly, cells were maintained in a 10% exosome-depleted FBS medium until the confluency was 70–80%, cells were subsequently washed three times with phosphate-buffered saline (PBS) and starved with serum-free medium for another 24 h. Then the supernatant was harvested and centrifuged at 2000 g for 15 min to remove dead cells. Next, the supernatant was collected again and centrifuged at 10000 g for 30 min at 4°C to remove cell debris. Finally, after centrifuging the supernatant at 120000 g for 70 min at 4°C with the Optima XPN-100 ultracentrifuge (Beckman Coulter, USA) followed by washing with PBS, the precipitation was collected and the final pellet containing exosome was resuspended in PBS.

### Transmission electron microscopy (TEM)

10 μL exosome suspension was added to a carbon-coated copper grid for 20 min. Then the grid was placed onto a drop with 1% glutaraldehyde for 5 min and further contrasted with 2% uranyl acetate for another 5 min. The grid was subsequently placed onto a drop with methylcellulose-uranyl acetate for 10 min and this step was completed on ice. A thin layer of methylcellulose membrane remained after gently sucking off the excess liquid with the filter paper. The copper grid was dried for several minutes and observed by using an HT7800 TEM (Hitachi, Japan) at 80 kV.

### Nanoparticle tracking analysis (NTA)

Exosome isolated from the supernatant was diluted in PBS, and to detect the particle size distribution and concentration of the exosomes, NTA was performed using the ZetaView PMX 110 (Particle Metrix, Germany) and ZetaView 8.04.02 software according to the manufacturer’s instructions.

### Western blot analysis

To measure exosomal surface markers, western blot analysis was conducted. Briefly, the exosome supernatant was denatured in sodium dodecyl sulfonate (SDS) buffer and then incubated with anti-CD81 (66866-1-Ig; Proteintech, USA), anti-TSG101 (14497-1-AP; Proteintech), and anti-HSP70 (66183-1-Ig; Proteintech).

### Exosomal RNA extraction, small RNA sequencing, and differential expression analysis

Total RNA, including miRNA, was extracted from exosomal samples using TRIzol Reagent (Invitrogen, USA) following the manufacturer’s protocol. The quality of RNA was analyzed by a NanoDrop 2000 (Thermo Fisher Scientific, USA) and Agilent Bioanalyzer 2100 (Agilent Technologies, USA). Library construction and sequencing of exo-miRNA were performed by Beijing Genomics Institute (BGI, China) as described previously [31]. The package DEGseq, a free R package, was used to identify DE-miRNAs from RNA-seq data. *P*-value < 0.05 and |log_2_FC (fold change)|>1 were chosen to identify DE-miRNAs as the cut-off criteria. A volcano plot was generated using the EnhancedVolcano (1.4.0) R package, and a heatmap of the DE-miRNAs was produced using the ‘heatmap’ package in R.

### Identification of the candidate exo-miRNAs

The database of exo-miRNAs related to BM in lung cancer was obtained from the published article published by Xiao-Rong Yang in 2021 with a total of 30 non-small cell lung cancer (NSCLC) patients including 16 BM+ patients and 14 BM- patients in this research [29]. The intersection of exosomal DE-miRNAs from the above-acquired database and our identified database from RNA-seq data was performed by the Venny 2.1 online web tool. The two databases identified the commonly shared DE-miRNAs as the candidate exo-miRNAs that were closely related to BM in lung cancer.

### Prediction of target mRNAs for the candidate exo-miRNAs

Three filters of miRWalk 2.0 database (http://mirwalk.umm.uni-heidelberg.de/), including TargetScan, miRDB, and miRTarbase, were used to predict the target mRNAs for the candidate exo-miRNAs. Only genes predicted by all three tools (TargetScan, miRDB, and miRTarbase) were needed for further analysis. The overlapped mRNAs by the three tools were considered as the candidate BM-associated target genes.

### Functional annotation and pathway enrichment analysis of the candidate exo-miRNAs

Based on the Kyoto Encyclopedia of Gene and Genome (KEGG) and Gene ontology (GO), the terms and pathway enrichment were then analyzed using the Database for Annotation, Visualization and Integrated Discovery (DAVID) online tools (https://david.ncifcrf.gov/tools.jsp). GO terms enrichment analysis was distributed into biological process (BP), cellular component (CC), and molecular function (MF), respectively. *p* < 0.05 was deemed as significant enrichment.

### Establishment of PPI network and module analysis

Search Tool for the Retrieval of Interacting Genes (STRING) online tool (https://www.stringdb.org/) was used to acquire the PPI network for the candidate mRNAs and only the interactions with a combined score >0.4 were considered to indicate a significant interaction. Subsequently, the PPI network was visualized with Cytoscape software (Version 3.9.1) [32]. Furthermore, the module analysis was carried out by the plug-in Molecular Complex Detection (MCODE, version 2.0.2). The criteria were defined as follows: degree cutoff = 2, node score cutoff = 0.2, k-core = 2, and max depth = 100.

### Hub gene identification, functional enrichment analysis, and miRNA-mRNA network construction

The hub genes were analyzed from the above key module network using the Cytohubba plug-in of Cytoscape (version 0.1) [33]. Genes with the cut-off criteria of degree ≥5 were regarded as hub genes [34]. Here, the top 10 genes with the largest degrees were considered as real hub genes. In addition, GO and KEGG pathway enrichment analysis of hub genes was carried out using the WebGestalt online tool (https://www.webgestalt.org/) with a threshold of false discovery rate (FDR) ≤0.05 [35]. Finally, the miRNA-hub mRNA regulatory network was constructed via Cytoscape software.

### Verification of the expression pattern of hub genes

The Sangerbox website (http://sangerbox.com/Tool) is a newly developed interactive web server, that provides interactive customizable analysis tools, including various kinds of expression analysis, pathway enrichment analysis, weighted correlation network analysis, and other common tools and functions, and offers a platform for researchers to analyze the RNA expression data from GEO, TCGA, ICGC, and other databases [36].

### Human protein atlas analysis

The Human Protein Atlas (http://www.proteinatlas.org) is an open-access website containing immunohistochemistry (IHC) data to allow researchers to investigate the expression patterns of different proteins expressed in various human tumors [37]. Patient information, staining intensity, staining location, and sample number of each type of cancer can be obtained. After screening, the expression of representative proteins in lung cancer and control normal tissues of the selected hub genes was evaluated by using IHC images.

### The cBioPortal for cancer genomics analysis

The cBioPortal for Cancer Genomics (cBioPortal, https://www.cbioportal.org/, version 5.0) is an open source for exploring, visualizing, and analyzing multidimensional cancer genomics data [38]. The frequency of genetic alterations in the identified hub genes in patients with lung cancer and their relationship to patient survival was examined by using cBioPortal.

### Gene set enrichment analysis (GSEA) of real hub genes

The GSEA analysis of real hub genes was further performed using the microarray dataset GSE175601 which was downloaded from the Gene Expression Omnibus (GEO) database of the NCBI database (https://www.ncbi.nlm.nih.gov/). The GSE175601 dataset was performed on the GPL21185 platform (Agilent-072363 SurePrint G3 Human GE v3 8x60K Microarray 039494), including three tumor tissues with bone metastases and three tumor tissues without bone metastases from patients with NSCLC [39]. The Sangerbox website was used to perform GSEA analysis to predict potential hallmarks [36]. A permutation test with 1,000 permutations was employed to screen the significantly changed pathways as previously described [40]. Thresholds of |NES| > 1 and Benjamini-Hochberg FDR <25% were considered statistically significant.

### Survival analysis

The Kaplan-Meier plotter online tool (https://kmplot.com/analysis/), a publicly available database, was used to evaluate the association between the identified hub genes expression and lung cancer patient survival. The overall survival (OS) curves were then generated, and the hazard ratio (HR) and its associated 95% confidence intervals (CI) and log-rank test *P* value were calculated. Log-rank test results with a *p*-value less than 0.05 were considered statistically significant.

### Statistical analysis

Statistical analyses were conducted using GraphPad Prism software (version 8.0; USA). According to each miRNA expression value, the receiver-operating characteristic (ROC) curve and the area under the ROC curve (AUC) were performed to estimate the sensitivity and specificity of each identified key exo-miRNA for the discriminating BM+ group from BM- group and the healthy controls. The AUC was calculated with 95% confidence intervals. *p* < 0.05 was considered significant.

### Availability of data and materials

Authors can provide all of the datasets on reasonable request.

## RESULTS

### Identification of candidate exo-miRNAs as biomarkers for BM in lung cancer

**Figure 1:**
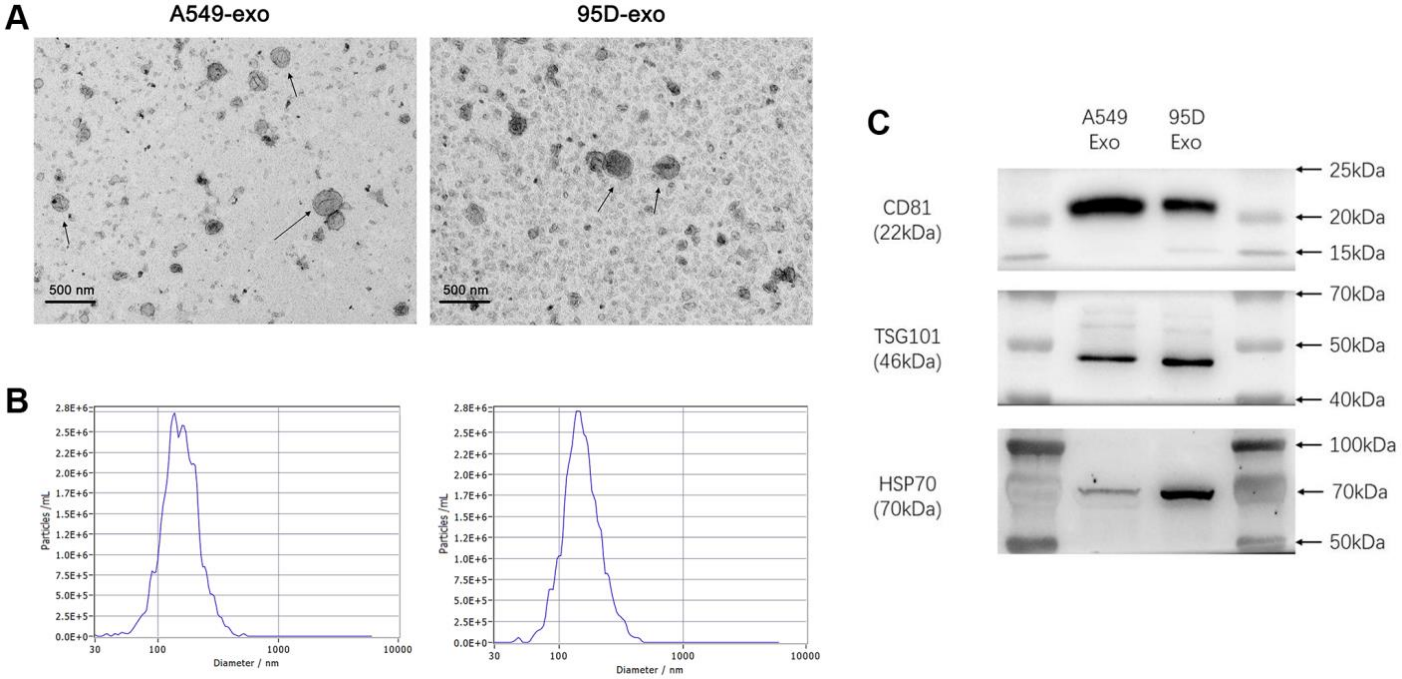
**Analysis of precipitated exosomes isolated from supernatants of cultured tumor cells.** (**A**) The morphology of exosomes was observed by TEM. (**B**) The exosomes were measured by NTA. (**C**) CD81, TSG101, and HSP70 were determined by western blot. Abbreviations: TEM: transmission electron microscopy; NTA: nanoparticle tracking analysis.

We used TEM, NTA, and western blot to characterize the isolated exosomes to determine the quality of exosomes from cell culture supernatants. TEM showed that the isolated exosomes were typically saucer-shaped vesicles with lipid bilayer membranes (Figure 1A). The NTA results revealed that the diameter of exosomes varied from 30 nm to 150 nm, and the exosomes from cell culture supernatants had a similar particle size distribution and peak area (Figure 1B). Western blot was used to detect the molecular markers for exosomes. As shown in Figure 1C, the exosomes from cell culture supernatants were found to be positive for exosome-associated markers such as CD81, TSG101, and HSP70. These results indicated that exosomes were successfully isolated from the cell culture supernatants.

**Figure 2:**
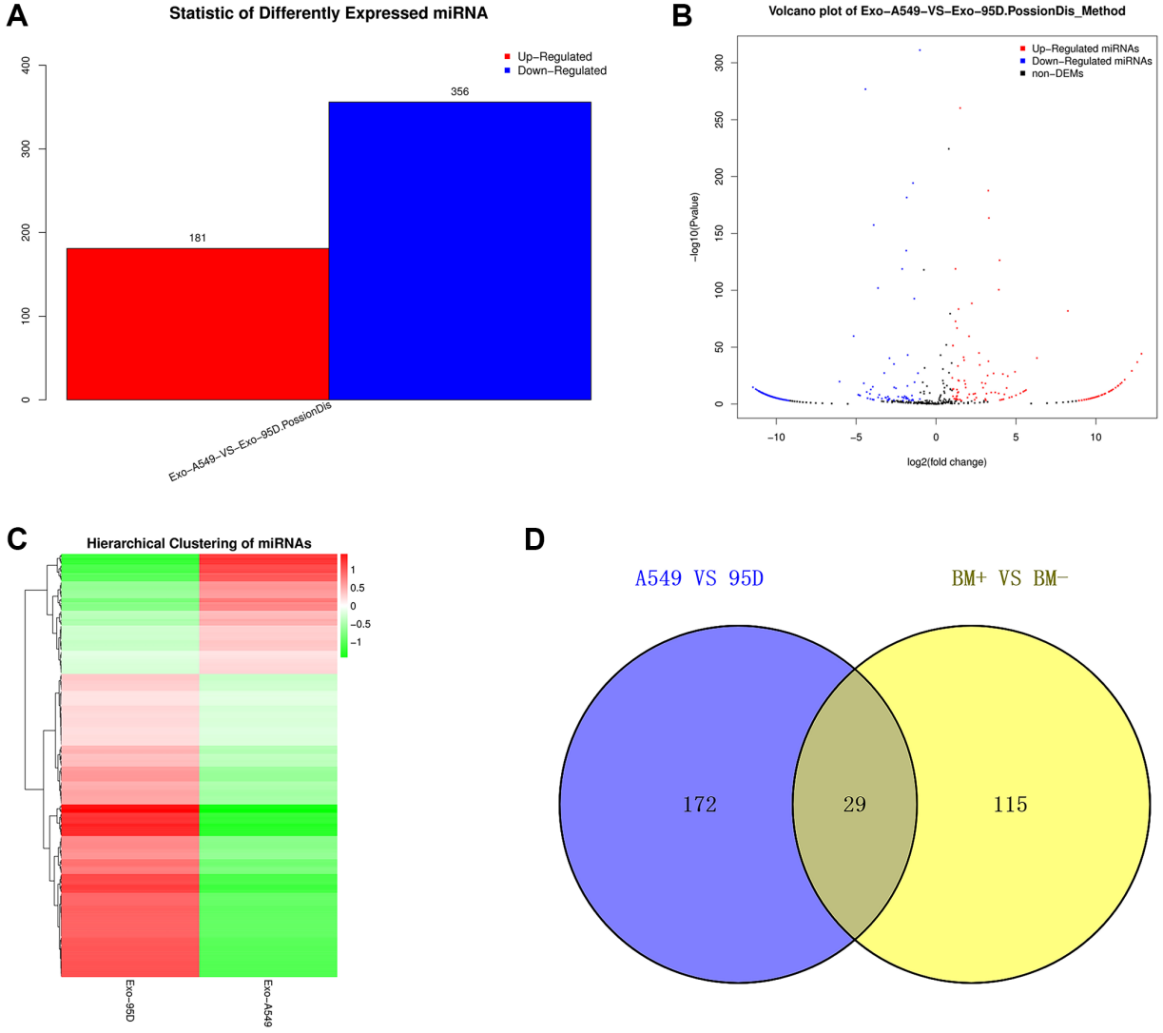
**Analysis of the DE-exo-miRNAs.** (**A**) Histogram showing the upregulated and downregulated DE-exo-miRNAs between the A549 and 95D cells. Red represented upregulated DE-exo-miRNAs; blue represented downregulated DE-exo-miRNAs. (**B**) The volcano map shows the distribution of DE-exo-miRNAs between A549 and 95D cells according to their *P* values and fold changes. Red dots represented upregulated DE-exo-miRNAs; blue dots represented downregulated DE-exo-miRNAs; black dots represented non-differentially-expressed miRNAs. (**C**) Heatmap showing DE-exo-miRNAs from A549 and 95D cells. The colors in the heatmap represented normalized gene expression values, with high expression values being colored in red and low expression values being colored in green. (**D**) The Venn diagram representing the 29 overlapping exo-miRNAs. Abbreviations: DE-exo-miRNAs: differentially expressed exosomal miRNAs; BM+: patients with bone metastasis; BM-: patients without bone metastasis.

We performed high-throughput sequencing to detect the miRNA expression of exosomes and screen DE-miRNAs between the highly-metastatic and lowly-metastatic groups. The results showed that a total of 556 known miRNAs and 1016 novel miRNAs were identified, and 537 miRNAs were found to be differentially expressed, among which 181 miRNAs were significantly upregulated and 356 downregulated (Figure 2A). Among the 556 known miRNAs, 144 miRNAs were significantly upregulated while 57 miRNAs were downregulated in the highly-metastatic group compared with the lowly-metastatic group. The volcano plots and heatmap of DE-miRNAs between the two groups were displayed in Figure 2B, 2C, respectively. Moreover, as shown in Supplementary Table 1, the top 10 DE-miRNAs were screened with a *p*-value less than 0.05.

To identify candidate exo-miRNAs related to BM in lung cancer, we intersected the data published by Xiao-Rong Yang in 2021 and our data obtained from small RNA sequencing. In total, we screened out 29 overlapping exo-miRNAs (Figure 2D and Table 1). These 29 miRNAs were considered as candidate exo-miRNAs.

**Table 1:** The 29 overlapping exosomal miRNAs identified in the study.

**miRNA ID**	**miRNA ID**	**miRNA ID**
hsa-miR-877-5p	hsa-miR-199a-5p	hsa-miR-1-3p
hsa-miR-505-3p	hsa-miR-197-3p	hsa-miR-23b-5p
hsa-miR-744-5p	hsa-miR-221-3p	hsa-miR-224-5p
hsa-miR-148b-3p	hsa-miR-23a-3p	hsa-let-7d-3p
hsa-miR-584-5p	hsa-miR-151a-3p	hsa-miR-133b
hsa-miR-641	hsa-miR-199a-3p	hsa-miR-340-3p
hsa-miR-340-5p	hsa-miR-199b-3p	hsa-miR-223-3p
hsa-miR-330-3p	hsa-miR-1307-3p	hsa-miR-222-3p
hsa-miR-425-3p	hsa-miR-937-3p	hsa-miR-21-5p
hsa-miR-28-5p	hsa-miR-133a-3p	

### Investigation of potential target genes of candidate exo-miRNAs

To elucidate the function of candidate miRNAs on the BM in lung cancer, the prediction of their target genes was performed using the miRWalk 2.0 database. To identify reliable target genes, the genes predicted by the 3 programs (TargetScan, miRDB, and miRTarbase) were identified as potential target genes of the 29 candidate exo-miRNAs. Finally, a total of 101 target genes of the 29 candidate exo-miRNAs were predicted (Supplementary Table 2).

**Figure 3:**
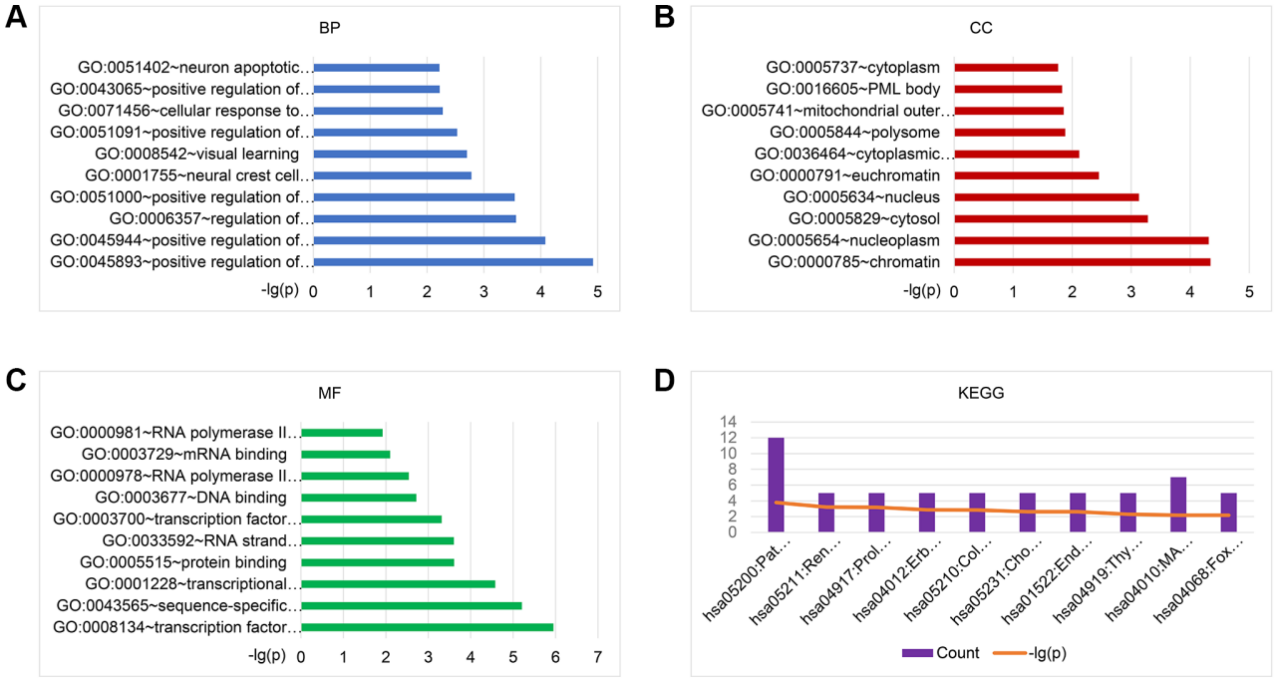
**GO and KEGG pathway enrichment analysis of the target genes of 29 exo-miRNAs using the DAVID database.** Top 10 significant terms of GO BP (**A**), CC (**B**), MF (**C**), and KEGG pathway (**D**) enrichment analysis of the target genes. Abbreviations: exo-miRNAs: exosomal miRNAs; BP: biological process; CC: cellular component; MF: molecular function.

To explore the biological functions of the 101 identified BM-associated target genes, we performed GO annotation and KEGG pathway enrichment analysis. Results showed that for the target genes of 29 candidate exo-miRNAs, according to BP, the top five main categories were positive regulation of transcription, DNA-templated, positive regulation of transcription from RNA polymerase II promoter, regulation of transcription from RNA polymerase II promoter, positive regulation of nitric-oxide synthase activity, and neural crest cell migration (Figure 3A and Supplementary Table 3). In terms of CC, the top five main categories were chromatin, nucleoplasm, cytosol, nucleus, and euchromatin (Figure 3B and Supplementary Table 3). For MF, the most represented categories were transcription factor binding, sequence-specific DNA binding, transcriptional activator activity, RNA polymerase II transcription regulatory region sequence-specific binding, protein binding, and RNA strand annealing activity (Figure 3C and Supplementary Table 3).

KEGG enrichment analysis was performed to identify pathways involved in BM in lung cancer. Results showed that for the target genes of the 29 candidate exo-miRNAs, KEGG pathways were mainly involved in pathways in cancer, renal cell carcinoma, prolactin signaling pathway, ErbB signaling pathway, and colorectal cancer (Figure 3D and Supplementary Table 3). Taken together, the above results collectively imply that the DE-exo-miRNAs may play key functional roles in the initiation and progression of BM in lung cancer by targeting their mRNAs.

### Construction of the exosomal miRNA-mRNA network and investigation of the network’s functional enrichment

**Figure 4:**
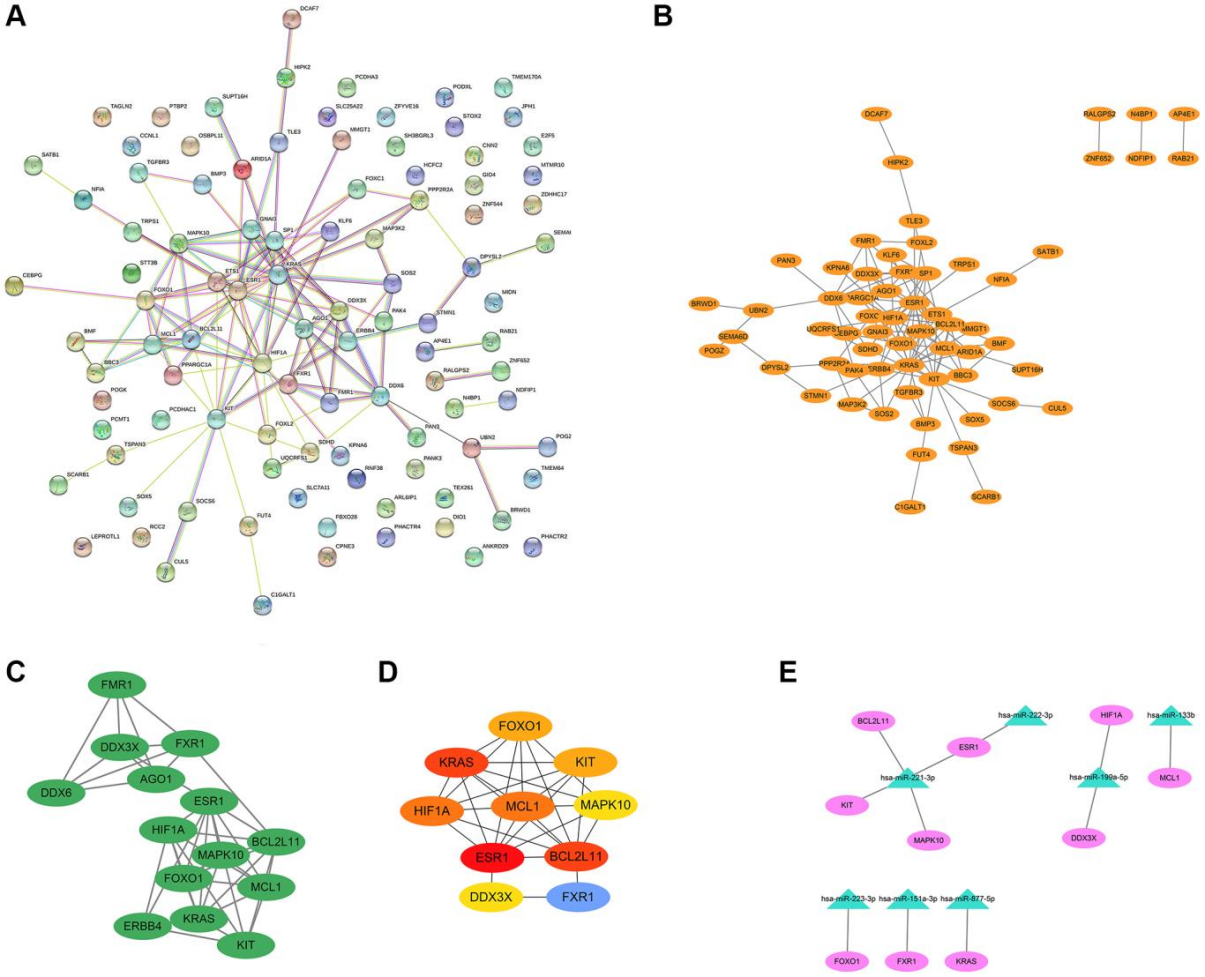
**PPI network, modular analysis, and miRNA-mRNA network construction.** The PPI network of 101 target genes of the 29 exo-miRNAs was analyzed using the STRING database (**A**) and Cytoscape software (**B**). (**C**) The key module was identified from the PPI network using the MCODE plug-in of Cytoscape. (**D**) The hub genes (degree: top 10) identified by the Cytohubba plug-in. (**E**) miRNA-hub gene network. In the miRNA-hub mRNA network, the pink ellipses represented hub genes, and the green triangles represented key miRNAs.

The PPI network of BM-related target genes was constructed using the STRING database. The results showed that a relevant PPI network was successfully constructed, which contained 100 nodes and 122 edges (Figure 4A). Next, this PPI network was visualized using the Cytoscape software (Figure 4B). Based on the entire network, the module analysis was further explored using the MCODE plug-in of Cytoscape. The results revealed that a significant module with a score ≥5 was identified from the network, which contained 14 nodes and 41 edges (Figure 4C). Subsequently, we applied the Cytohubba plug-in of Cytoscape to screen out the top 10 hub genes of the network using the degree method. In the PPI network, the hub genes were ESR1, KRAS, BCL2L11, MCL1, HIF1A, FOXO1, KIT, FXR1, MAPK10, and DDX3X (Figure 4D and Table 2). Finally, the miRNA-hub gene network was established by Cytoscape software. As shown in Figure 4E and Table 3, an exosomal miRNA-mRNA network consisted of 7 key miRNAs (hsa-miR-221-3p, hsa-miR-222-3p, hsa-miR-199a-5p, hsa-miR-133b, hsa-miR-223-3p, hsa-miR-151a-3p, and hsa-miR-877-5p) and 10 hub mRNAs (ESR1, KIT, MAPK10, BCL2L11, HIF1A, DDX3X, MCL1, FOXO1, FXR1, and KRAS) was constructed. These miRNAs and mRNAs were respectively regarded as key exo-miRNAs and hub mRNAs that may play crucial roles in BM of lung cancer via exosomes.

**Table 2:** Ten hub genes identified in the key module using the degree method.

**Rank**	**Genes**	**Description**	**Score**
1	ESR1	Estrogen Receptor 1	9
2	KRAS	KRAS Proto-Oncogene, GTPase	8
2	BCL2L11	BCL2 Like 11	8
4	MCL1	MCL1 Apoptosis Regulator, BCL2 Family Member	7
4	HIF1A	Hypoxia Inducible Factor 1 Subunit Alpha	7
6	FOXO1	Forkhead Box O1	6
6	KIT	KIT Proto-Oncogene, Receptor Tyrosine Kinase	6
8	FXR1	FMR1 Autosomal Homolog 1	5
8	MAPK10	Mitogen-Activated Protein Kinase 10	5
8	DDX3X	DEAD-Box Helicase 3 X-Linked	5

**Table 3:** The list of the 7 hub exosomal miRNAs.

**miRNA**	**mRNA Count**	**mRNA**
hsa-miR-221-3p	4	ESR1, MAPK10, KIT, BCL2L11
hsa-miR-199a-5p	2	HIF1A, DDX3X
hsa-miR-222-3p	1	ESR1
hsa-miR-133b	1	MCL1
hsa-miR-223-3p	1	FOXO1
hsa-miR-151a-3p	1	FXR1
hsa-miR-877-5p	1	KRAS

**Figure 5:**
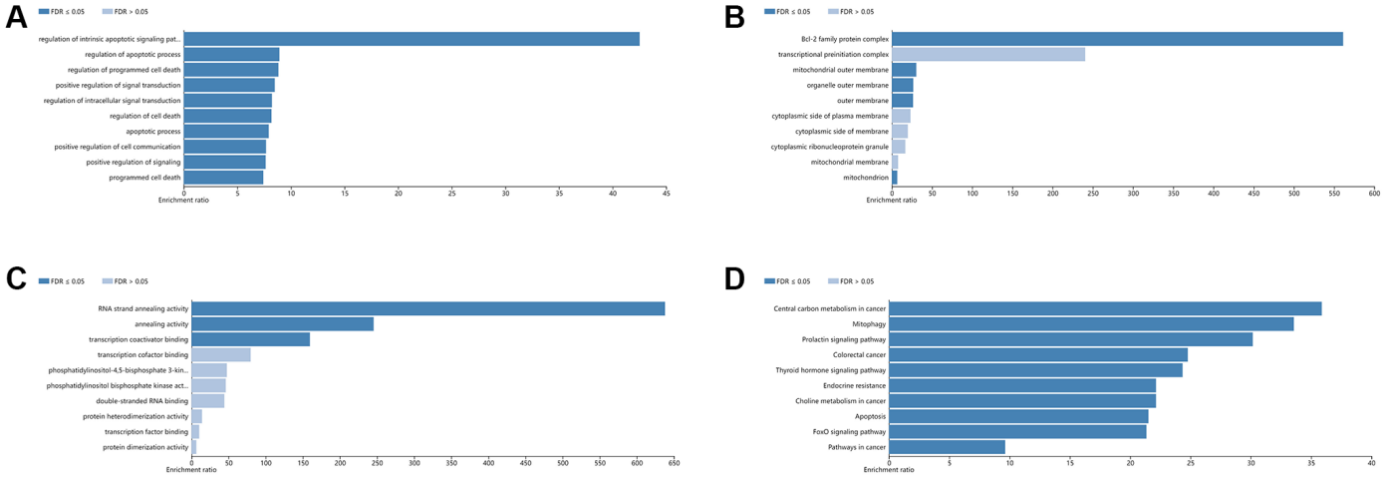
**GO and KEGG analysis of hub genes using the WebGestalt database.** Top 10 significant terms of GO BP (**A**), CC (**B**), MF (**C**), and KEGG pathway (**D**) enrichment analysis. Abbreviations: BP: biological process; CC: cellular component; MF: molecular function.

GO and KEGG pathway analyses for the identified 10 hub genes were then performed using the Webgestalt database. The results indicated that in terms of BP, the top 5 were regulation of the intrinsic apoptotic signaling pathway, regulation of the apoptotic process, regulation of programmed cell death, positive regulation of signal transduction, and regulation of intracellular signal transduction (Figure 5A). According to CC, the top 5 were the Bcl-2 family protein complex, mitochondrial outer membrane, organelle outer membrane, outer membrane, and mitochondrion (Figure 5B). For the analysis of MF, the identified 10 hub genes were enriched in RNA strand annealing activity, annealing activity, and transcription coactivator binding (Figure 5C). KEGG analysis revealed that the top 5 pathways identified were central carbon metabolism in cancer, mitophagy, prolactin signaling pathway, colorectal cancer, and thyroid hormone signaling pathway (Figure 5D).

### Validation of the hub genes and survival analysis

**Figure 6:**
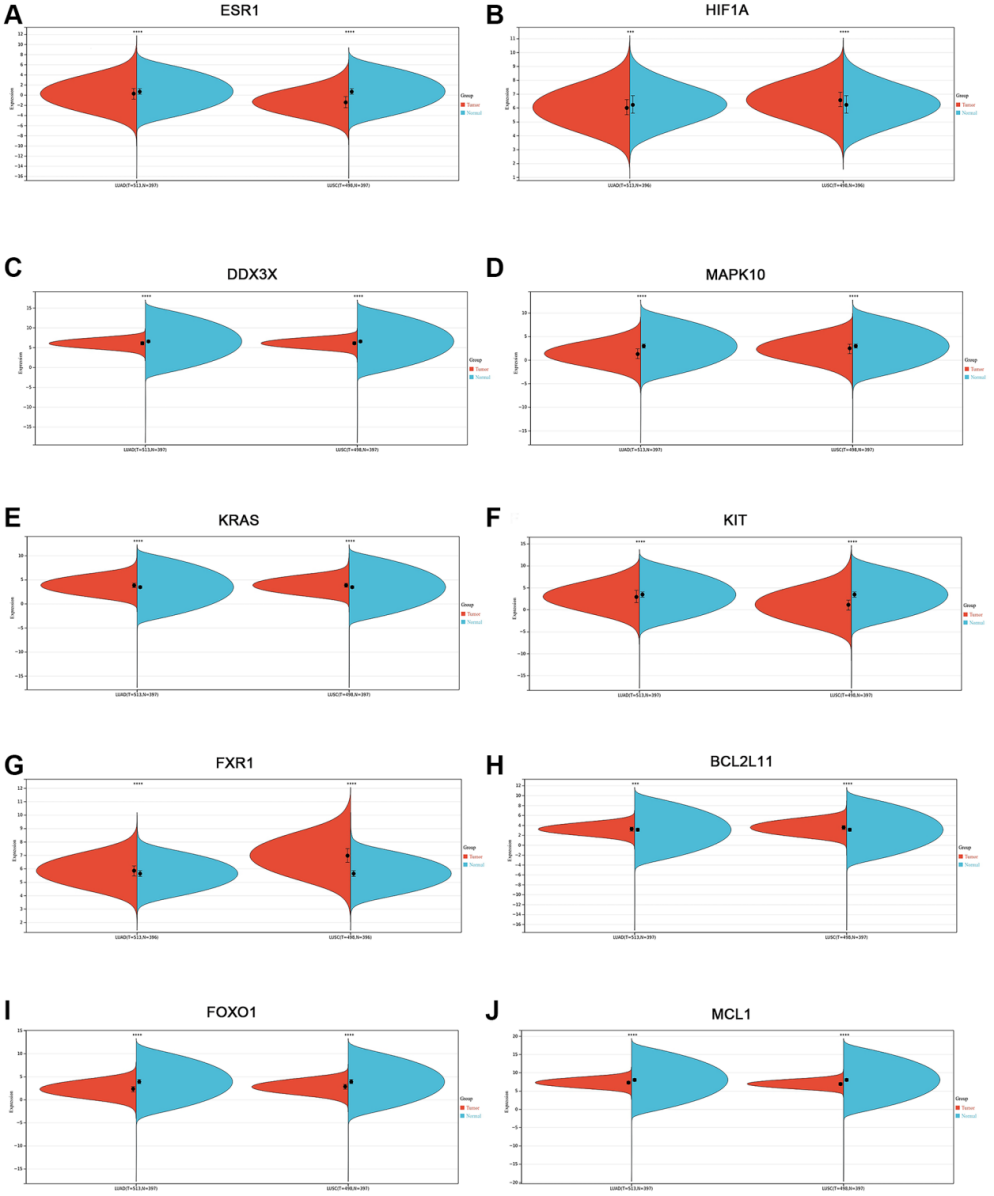
**Validation of the mRNA expression level of 10 hub genes in lung cancer using the Sangerbox website database.** (**A**) ESR1. (**B**) HIF1A. (**C**) DDX3X. (**D**) MAPK10. (**E**) KRAS. (**F**) KIT. (**G**) FXR1. (**H**) BCL2L11. (**I**) FOXO1. (**J**) MCL1. ^*^p < 0.05; ^**^p < 0.01; ^***^p < 0.0001.

**Figure 7:**
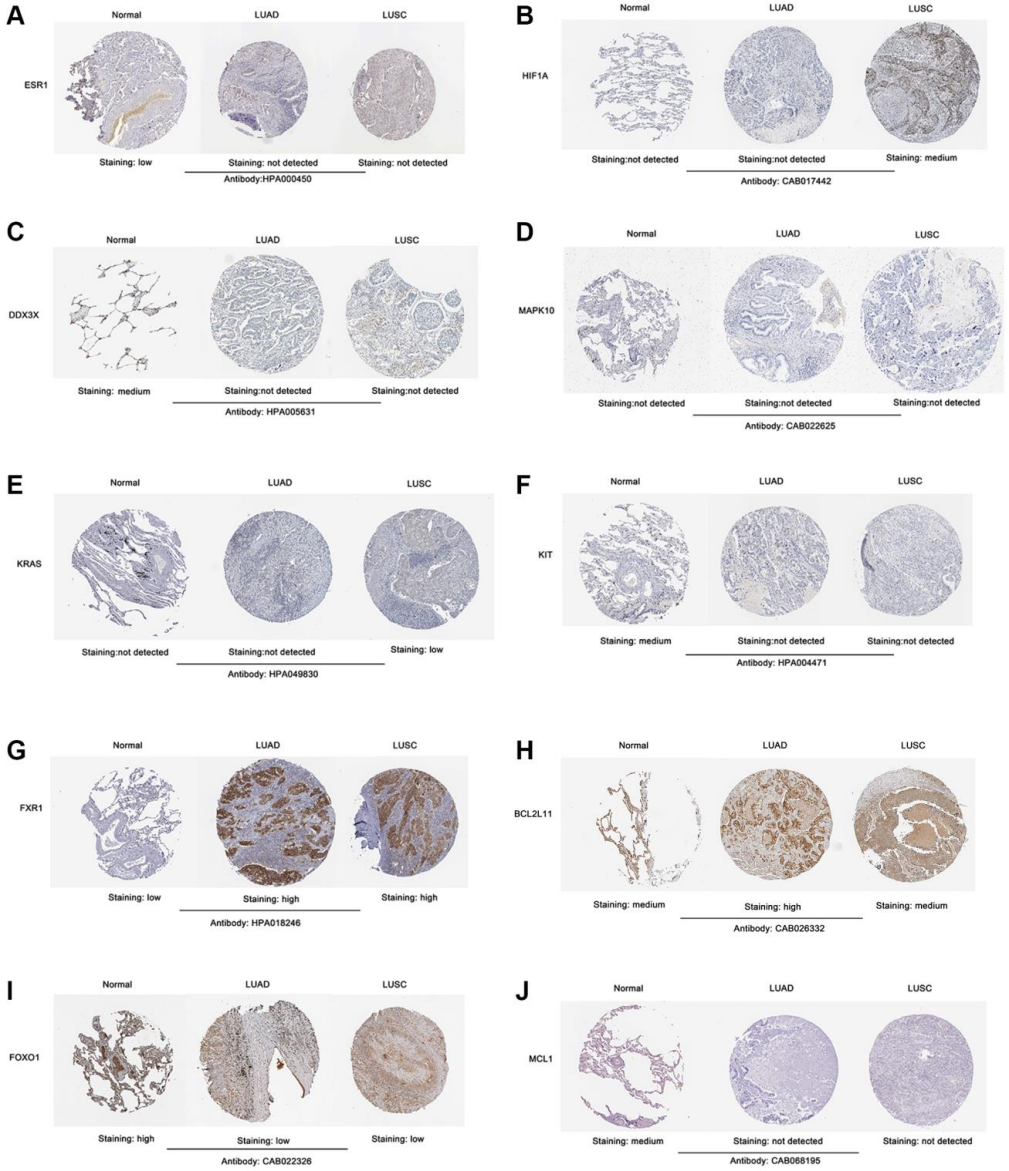
**Validation of the protein expression level of screened 10 hub genes in lung cancer samples according to the IHC images in The Human Protein Atlas database.** (**A**) ESR1. (**B**) HIF1A. (**C**) DDX3X. (**D**) MAPK10. (**E**) KRAS. (**F**) KIT. (**G**) FXR1. (**H**) BCL2L11. (**I**) FOXO1. (**J**) MCL1. Abbreviation: IHC: Immunohistochemistry.

To confirm the expression level of the identified 10 hub genes in lung cancer, we used mRNA expression data from the Sangerbox website database. The results showed that the mRNA expression levels of ESR1 (Figure 6A), DDX3X (Figure 6C), MAPK10 (Figure 6D), KIT (Figure 6F), FOXO1 (Figure 6I), and MCL1 (Figure 6J) were significantly lower in both lung adenocarcinoma (LUAD) and lung squamous cell carcinoma (LUSC) tissues (*p* < 0.001) than normal lung tissues. The mRNA expression levels of KRAS (Figure 6E), FXR1 (Figure 6G), and BCL2L11 (Figure 6H) were markedly upregulated in both LUAD and LUSC tissues (*p* < 0.001) compared with those in normal lung tissues. Interestingly, the HIF1A mRNA expression level of LUAD tissues was significantly lower than that of normal lung tissues (*p* < 0.001) (Figure 6B). In contrast, the mRNA expression level of HIF1A was markedly increased in LUSC tissues, compared with the normal lung tissues (*p* < 0.001) (Figure 6B). Next, we used the Human Protein Atlas to assess the protein expression levels of the identified 10 hub genes in lung cancer. The results showed that the protein expression levels of the identified 10 hub genes (ESR1, HIF1A, DDX3X, MAPK10, KRAS, KIT, FXR1, BCL2L11, FOXO1, and MCL1) displayed similar patterns of changes as the mRNA expression levels (Figure 7A–7J).

**Figure 8:**
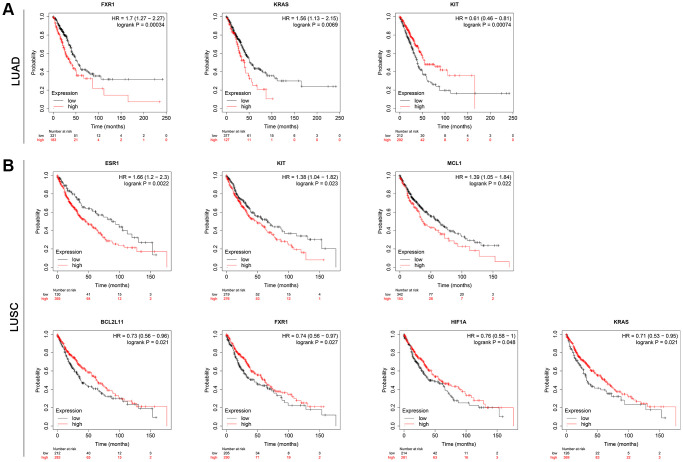
**Results for the OS analysis of the hub mRNAs in lung cancer patients based on the Kaplan-Meier plotter online database.** (**A**) LUAD. (**B**) LUSC. Abbreviations: OS: overall survival; LUAD: Lung adenocarcinoma; LUSC: Lung squamous cell carcinoma.

To further validate the 10 hub genes, their prognostic significance in lung cancer patients was subsequently analyzed by the Kaplan-Meier plotter online database. The survival analysis included a total of 513 LUAD patients and 501 LUSC patients. As shown in Figure 8A, among 10 hub genes, a higher expression of FXR1 and KRAS was related to a worse overall survival (OS) in LUAD patients. Interestingly, a higher KIT expression was significantly associated with better OS (*p* = 0.00074) in LUAD patients. However, there was no statistically significant difference in OS between high-expression and low-expression of ESR1, HIF1A, DDX3X, MAPK10, BCL2L11, FOXO1, and MCL1 in LUAD patients (Supplementary Figure 1A). For LUSC patients, increased expression of ESR1, KIT, and MCL1 predicted a poorer OS, while increased expression of BCL2L11, FXR1, HIF1A, and KRAS was associated with a favorable OS (Figure 8B). Nevertheless, there was no statistically significant difference in OS between high-expression and low-expression of DDX3X, MAPK10, and FOXO1 in LUSC patients (Supplementary Figure 1B). These data imply that high expression of the identified seven real hub genes (FXR1, KRAS, ESR1, KIT, MCL1, BCL2L11, and HIF1A) may play important roles in the carcinogenesis or progression of lung cancer.

### Genetic alterations and GSEA of real hub genes

**Figure 9:**
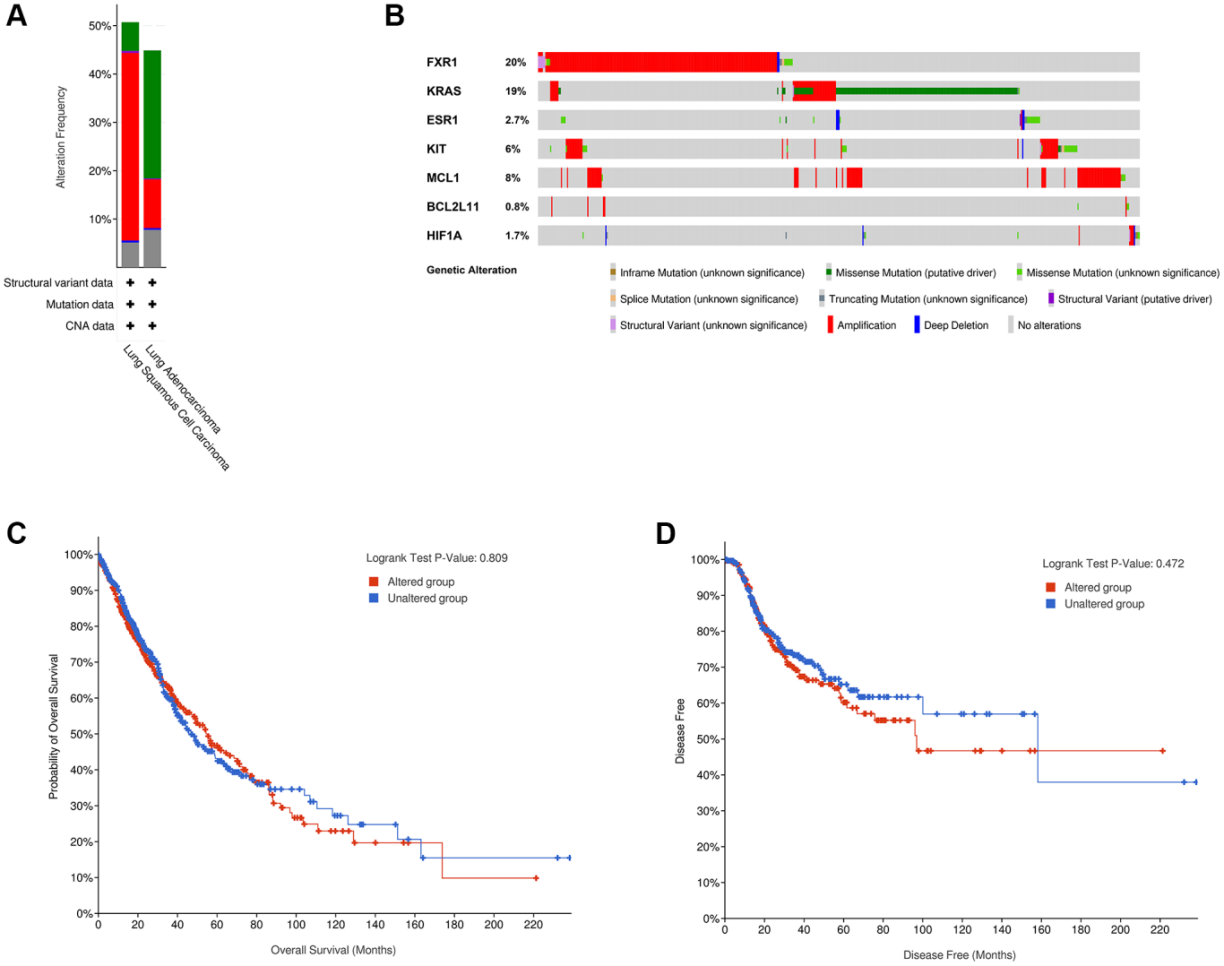
**Genetic alterations linked to 7 real hub genes in lung cancer in cBioPortal online database.** (**A**) Seven hub gene alterations in LUAD (TCGA, PanCancer Atlas) and LUSC (TCGA, PanCancer Atlas). (**B**) Alteration frequencies of seven hub genes based on the LUAD (TCGA, PanCancer Atlas) and LUSC (TCGA, PanCancer Atlas). Kaplan-Meier plots showing OS (**C**) and DFS (**D**) in cases with and without hub gene alterations. Abbreviations: LUAD: Lung adenocarcinoma; LUSC: Lung squamous cell carcinoma; OS: overall survival; DFS: disease-free survival.

To analyze the genetic alterations of the 7 real hub genes in lung cancer in the cBioPortal online database, the LUAD (TCGA, PanCancer Atlas) and LUSC (TCGA, PanCancer Atlas) databases were used. Results showed that the percentages of genetic alterations in the two datasets were 44.88% (254/566) and 50.72% (247/487), respectively (Figure 9A). The predominant alterations were mutations for LUAD, however, for LUSC, the predominant alterations were amplifications instead of mutations (Figure 9A). Specific to lung cancer, the alteration frequency of the seven hub genes was 20% for FXR1, 19% for KRAS, 2.7% for ESR1, 6% for KIT, 8% for MCL1, 0.8% for BCL2L11, and 1.7% for HIF1A (Figure 9B). Besides, the analysis of the correlation between cases with hub gene alterations and survival outcomes was also performed. However, results showed that cases with hub gene alterations were not statistically significant (*p* = 0.809 for OS and *p* = 0.472 for disease-free survival) (Figure 9C, 9D).

**Figure 10:**
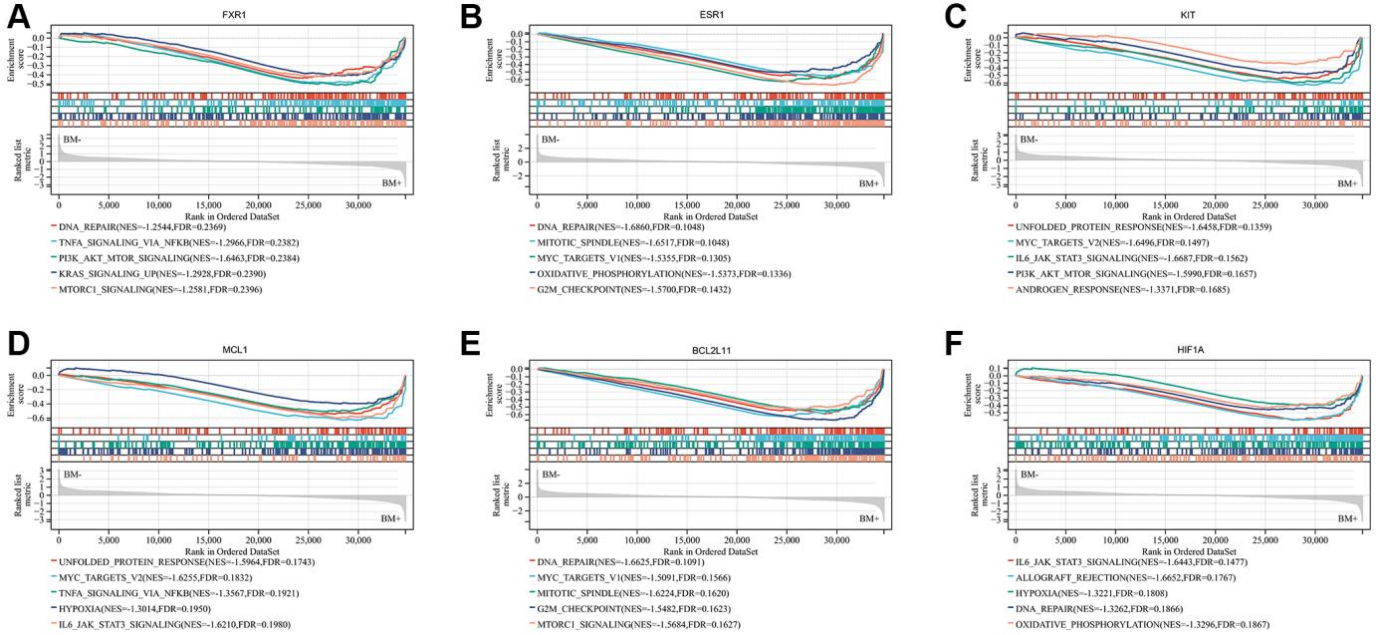
**Significant genes related to six real hub genes and hallmark pathways in lung cancers were obtained by GSEA based on the Sangerbox website database using GSE175601.** Top five gene sets according to a GSEA enrichment score for FXR1 (**A**), ESR1 (**B**), KIT (**C**), MCL1 (**D**), BCL2L11 (**E**), and HIF1A (**F**). Abbreviation: GSEA: Gene set enrichment analysis.

To further investigate the potential function of the seven real hub genes involved in the progression of lung cancer, GSEA analysis was performed based on the microarray dataset GSE175601. GSEA was employed to perform a hallmark gene sets analysis for FXR1, KRAS, ESR1, KIT, MCL1, BCL2L11, and HIF1A. FXR1 (Figure 10A), ESR1 (Figure 10B), BCL2L11 (Figure 10E), and HIF1A (Figure 10F) were all enriched in DNA repair. KIT (Figure 10C), MCL1 (Figure 10D), and HIF1A (Figure 10F) were all enriched in IL6/JAK/STAT3 signaling. ESR1 (Figure 10B) and BCL2L11 (Figure 10E) were all enriched in the G_2_/M checkpoint, mitotic spindle, and MYC targets v1. KIT (Figure 10C) and MCL1 (Figure 10D) were all enriched in MYC targets v2 and unfolded protein response. FXR1 and KIT were all enriched in PI3K/AKT/ mTOR signaling. FXR1 (Figure 10A) and MCL1 (Figure 10D) were all enriched in TNFα signaling via NF-kB. FXR1 (Figure 10A) and BCL2L11 (Figure 10E) were all enriched in mTORC1 signaling. ESR1 (Figure 10B) and HIF1A (Figure 10F) were all enriched in oxidative phosphorylation. MCL1 (Figure 10D) and HIF1A (Figure 10F) were all enriched in hypoxia. However, GSEA analysis of KRAS showed that there was no statistical significance according to the values of |NES| and FDR (Supplementary Figure 2). Some of the above-mentioned pathways have been demonstrated to be closely related to BM of lung cancer. Noticeably, these pathways were significantly involved in bone metastatic samples.

### Assessment of the diagnostic significance of the key exosomal miRNAs in the BM of lung cancer

**Figure 11:**
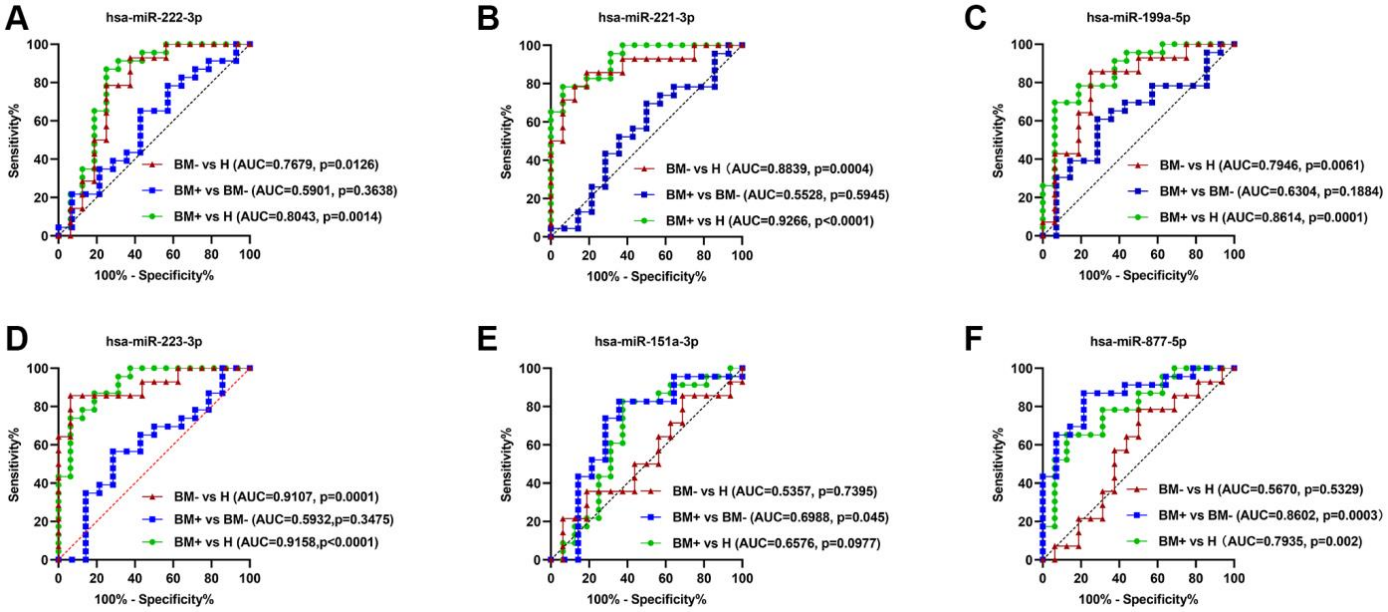
**ROC curve analysis of the six key exo-miRNAs.** (**A**) hsa-miR-222-3p. (**B**) hsa-miR-221-3p. (**C**) hsa-miR-199ª-5p. (**D**) hsa-miR-223-3p. (**E**) hsa-miR-151ª-3p. (**F**) hsa-miR-877-5p. The ROC curves to discriminate the BM- group from healthy controls (H) in the validation set are marked by red lines; the ROC curves to differentiate the BM+ group from the BM- group in the validation set are marked by blue lines; the ROC curves to differentiate the BM+ group from H in the validation set are marked by green lines. Abbreviations: ROC: receiver operator characteristic curve; BM+: patients with bone metastasis; BM−: patients without bone metastasis.

ROC curve analysis was performed using the GraphPad Prism software to estimate the accuracy of identified seven key exo-miRNAs in discriminating the BM+ group from the BM- group and healthy controls. The AUCs, sensitivity, and specificity were calculated. hsa-miR-222-3p (Figure 11A), hsa-miR-221-3p (Figure 11B), hsa-miR-199a-5p (Figure 11C), and hsa-miR-223-3p (Figure 11D) could be potential biomarkers to either distinguish the BM- group from healthy controls, with the AUCs of 0.7679 (*p* = 0.0126), 0.8839 (*p* = 0.0004), 0.7946 (*p* = 0.0061), and 0.9107 (*p* = 0.0001) or distinguish the BM+ group from healthy controls, with the AUCs of 0.8043 (*p* = 0.0014), 0.9266 (*p* < 0.0001), 0.8614 (*p* = 0.0001), and 0.9158 (*p* < 0.0001). Importantly, among the identified 7 key exo-miRNAs, only hsa-miR-151a-3p (Figure 11E) and hsa-miR-877-5p (Figure 11F) could distinguish the BM+ group from the BM- group, with AUCs of 0.6988 (*p* = 0.045) and 0.8602 (*p* = 0.0003), respectively. hsa-miR-133b could only distinguish the BM+ group from healthy controls, with AUCs of 0.7364 (*p* = 0.013) (Supplementary Figure 3). These data indicated that exosomal hsa-miR-151a-3p and hsa-miR-877-5p have relatively high diagnostic accuracy in discriminating the BM+ group from the BM- group and can serve as the non-invasive novel biomarkers for detecting BM in lung cancer.

## DISCUSSION

Lung cancer is one of the most common malignancies in the world and the main cause of cancer-related deaths [1, 2]. Lung cancer frequently metastasizes to bone resulting in severe SREs, which greatly decrease the quality of life and OS rate of the patients [3]. Although the treatment of BM in lung cancer has made great progress over recent years, patients’ prognosis remains poor [7, 41]. Hence, early diagnosis of BM in lung cancer may be an effective method to reduce the morbidity and mortality related to the disease. Accumulating evidence has shown that exosomes play crucial roles in the metastasis of lung cancer cells [42–44]. Therefore, exosomes have the potential to be explored for diagnostic, prognostic, and therapeutic applications [26]. Recently, in several studies, exo-miRNAs were found to be new biomarkers of BM in lung cancer [45, 46]. However, the underlying mechanism of exosome-regulated lung cancer progression has not been fully revealed. Here, we aimed to explore exo-miRNAs as potential early diagnostic biomarkers for predicting BM in lung cancer and uncover the underlying mechanism involved in BM of lung cancer by performing bioinformatical analysis.

In this study, we first investigated the exo-miRNA profiles derived from highly-metastatic and lowly-metastatic lung cancer cell lines by small RNA sequencing. Combined with a comprehensive analysis of the open-access exo-miRNA data for BM in lung cancer, as a result, we identified 29 candidate exo-miRNAs that were considered to be closely related to BM in lung cancer. Using the miRWalk 2.0 database, we identified 101 target genes of the 29 candidate exo-miRNAs. Additionally, GO and KEGG analysis indicated that the screened target genes are related to the pathogenesis of BM in lung cancer. A relevant PPI network consisting of 100 nodes and 122 edges was constructed and visualized via the STRING database and Cytoscape software. A significant module was screened by the MCODE plug-in of Cytoscape software. Furthermore, based on the module, 10 hub genes with degrees ≥10 were identified. Finally, we constructed an exosomal miRNA-mRNA network, including 7 key miRNAs (hsa-miR-221-3p, hsa-miR-222-3p, hsa-miR-199a-5p, hsa-miR-133b, hsa-miR-223-3p, hsa-miR-151a-3p, and hsa-miR-877-5p) and 10 hub mRNAs (ESR1, KIT, MAPK10, BCL2L11, HIF1A, DDX3X, MCL1, FOXO1, FXR1, and KRAS). It is worth noticing that the results of most previous studies on the above-mentioned 7 key miRNAs are consistent with our analysis. For instance, Yin et al. found that hsa-miR-221-3p promoted the cell growth of NSCLC by targeting p27 [47]. Chen et al. found that hsa-miR-222-3p promoted cell proliferation and inhibited apoptosis by targeting PUMA (BBC3) in NSCLC [48]. Several studies have reported that hsa-miR-199a-5p is involved in the progression of lung cancer [49–51]. It was confirmed that hsa-miR-133b targeted NCAPH to promote β-catenin degradation and reduced cancer stem cell maintenance in NSCLC [52]. Luo et al. found hsa-miR-223-3p functioned as a tumor suppressor in LUSC by miR-223-3p-mutant p53 regulatory feedback loop [53]. It was reported that altered expression of hsa-miR-151a-3p was related to activation of divergent biological pathways in lung cancer cells [54]. hsa-miR-877-5p was reported to be involved in the carcinogenesis of lung cancer [55, 56]. Taken together, these results suggest that the identified 7 key exo-miRNAs may play important roles in the development of lung cancer.

The 10 hub genes including ESR1, KIT, MAPK10, BCL2L11, HIF1A, DDX3X, MCL1, FOXO1, FXR1, and KRAS were screened from PPI network analysis. According to the Sangerbox platform and the Human Protein Atlas database, abnormal expressions of the above-mentioned hub genes were found in lung cancer tissues. Functional annotation analysis showed that the 10 hub genes significantly enriched in GO terms were related to apoptosis and RNA transcription, such as regulation of intrinsic apoptotic signaling pathway, regulation of apoptotic process, regulation of programmed cell death, Bcl-2 family protein complex, and RNA strand annealing activity, which are implicated in the regulation of cell growth in lung cancer [57, 58]. Additionally, the KEGG pathway enrichment analysis revealed that the 10 hub genes were mainly enriched in central carbon metabolism in cancer, mitophagy, prolactin signaling pathway, colorectal cancer, and thyroid hormone signaling pathway, which were involved in tumorigenesis and tumor progression of many cancers, including lung cancer [59, 60].

Subsequently, to further evaluate the prognostic significance of the 10 hub genes in lung cancer, we conducted a Kaplan-Meier survival analysis. As a result, 7 real hub genes came out of this analysis: FXR1, KRAS, ESR1, KIT, MCL1, BCL2L11, and HIF1A. This finding suggests that the 7 real hub genes can be used to predict the prognosis of patients with lung cancer. Further gene mutation analysis confirmed that the percentages of gene alterations of the 7 real hub genes were 44.88% in LUAD and 50.72% in LUSC, respectively. Almost every hub gene had different types of genetic alterations, and we found that amplification, missense mutation, and deep deletion were the three most common types of aberrations. These findings imply that genetic alterations of the 7 real hub genes may play important roles in lung cancer initiation and progression.

As reported before, Qian et al. found that FXR1 is a key regulator of tumor progression, and its overexpression is critical to the growth of NSCLC cells [61]. Multiple studies have shown that KRAS is the most common mutant oncogene in NSCLC, and KRAS mutant lung cancer has a poor prognosis and is resistant to chemotherapy; in addition, in the presence of this mutation, patients are more likely to have liver and brain metastasis [62–64]. It has been suggested that higher ESR1 expression is correlated with worse OS in lung cancer patients [65]. Several studies have indicated that KIT is associated with the development of lung cancer. For example, Funkhouser et al. found KIT mutation is more likely to cause brain metastasis in NSCLC [66]. Zhou et al. found targeting c-KIT can inhibit the growth and invasion of gefitinib-resistant NSCLC cells by reducing cancer stemness, EMT, and acquired drug resistance [67]. In lung cancer, MCL1 has been suggested to play a key role in cancer stem cells, including invasion, chemotherapy resistance, and tumorigenesis [68]. In NSCLC patients with EGFR mutations, the BIM deletion polymorphism results in an inherent resistance or reduced sensitivity to EGFR TKIs [69]. Evidence suggests that HIF1A drives tumor progression via regulating glycolysis, angiogenesis, and cell cycle progression in lung cancer [70–72]. In summary, these findings were consistent with our results, indicating that these 7 real hub genes may play critical roles in the pathogenesis and progression of lung cancer. Therefore, these 7 real hub genes have potential as candidate diagnostic biomarkers and prognostic predictors for lung cancer.

Moreover, GSEA was used to further investigate the biological functions of the 7 real hub genes in lung cancer and the results indicated that the high expression groups of FXR1, ESR1, KIT, MCL1, BCL2L11, and HIF1A were significantly enriched in the pathways associated with cell proliferation, such as IL6/JAK/STAT3 and PI3K/AKT/mTOR signaling pathway. Since the specific functions of these hub genes in lung cancer are not yet clear, the potential molecular mechanisms need to be further studied.

In addition, among the 7 key exo-miRNAs, ROC analysis showed that only hsa-miR-151a-3p and hsa-miR-877-5p have good predictive ability to distinguish between BM+ group and BM- group, with the corresponding AUCs were 0.6988 (*p* = 0.045) and 0.8602 (*p* = 0.0003), respectively. To the best of our knowledge, there are no reports for the diagnostic value of the two miRNAs in the predicting BM in lung cancer, suggesting hsa-miR-151a-3p and hsa-miR-877-5p may serve as novel non-invasive diagnostic markers for predicting BM in lung cancer.

Our current research has several limitations. Firstly, the sample size obtained from the publicly available data was small. Therefore, the study findings need to be further confirmed in large clinical samples. Secondly, the expressions of screened 7 key exo-miRNAs and 7 real hub genes have not been further validated with RT-qPCR experiments. Thirdly, the present study is chiefly concerned with assessing the diagnostic value of hsa-miR-151a-3p and hsa-miR-877-5p. Nevertheless, in the future, we need to carry out *in vitro* and *in vivo* experiments to verify the functions and mechanisms of the two miRNAs in BM in lung cancer.

## CONCLUSIONS

The study identified DE-exo-miRNAs and mRNAs linked to BM in lung cancer, constructing an exosomal miRNA-mRNA network. Key miRNAs and hub genes involved in BM were identified, with exosomal hsa-miR-151a-3p and hsa-miR-877-5p being the most promising biomarkers. These findings provide new insights for diagnosing and treating BM in lung cancer.

## Supplementary Materials

Supplementary Figures

Supplementary Tables
