# Supplementary material for: Exosomal hsa-miR-151a-3p and hsa-miR-877-5p are potential novel biomarkers for predicting bone metastasis in lung cancer
Source: Aging (Albany NY). 2023 Dec 18;15(24):14864–88. doi: 10.18632/aging.205314 (PMC10781484; doi:10.18632/aging.205314)
Supplement: Supplementary Figures [file aging-15-205314-s001.pdf]

SUPPLEMENTARY FIGURES

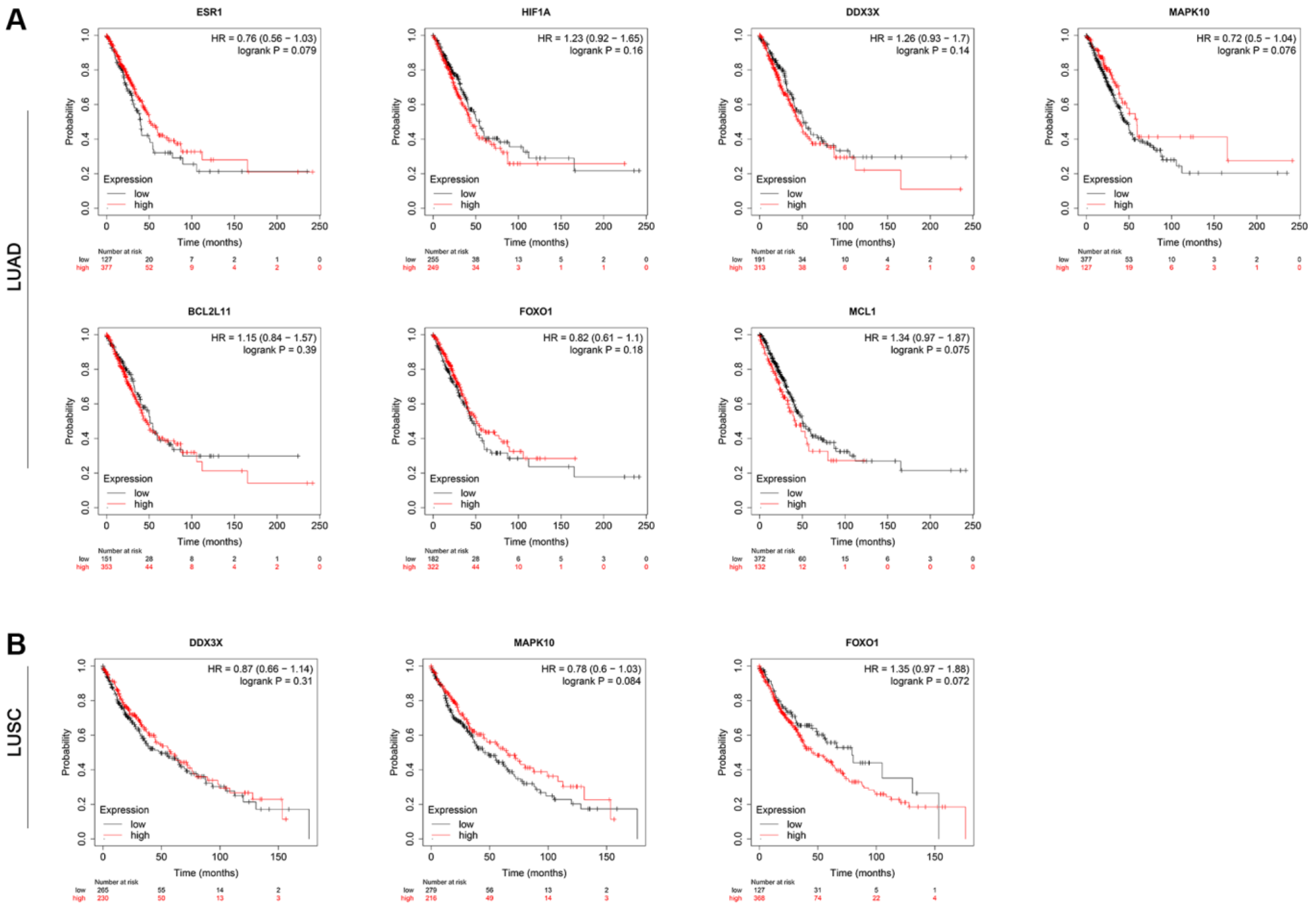

Supplementary Figure 1. Kaplan-Meier curves for hub mRNAs in lung cancer derived from TCGA data in the Kaplan-Meier plotter database. OS was not substantially linked with any of the seven hub genes (ESR1, HIF1A, DDX3X, MAPK10, BCL2L11, FOXO1, and MCL1). The red line shows individuals with high expression, whereas the black line shows those with low expression. (**A**) LUAD. (**B**) LUSC. Abbreviations: OS: overall survival; LUAD: Lung adenocarcinoma; LUSC: Lung squamous cell carcinoma.

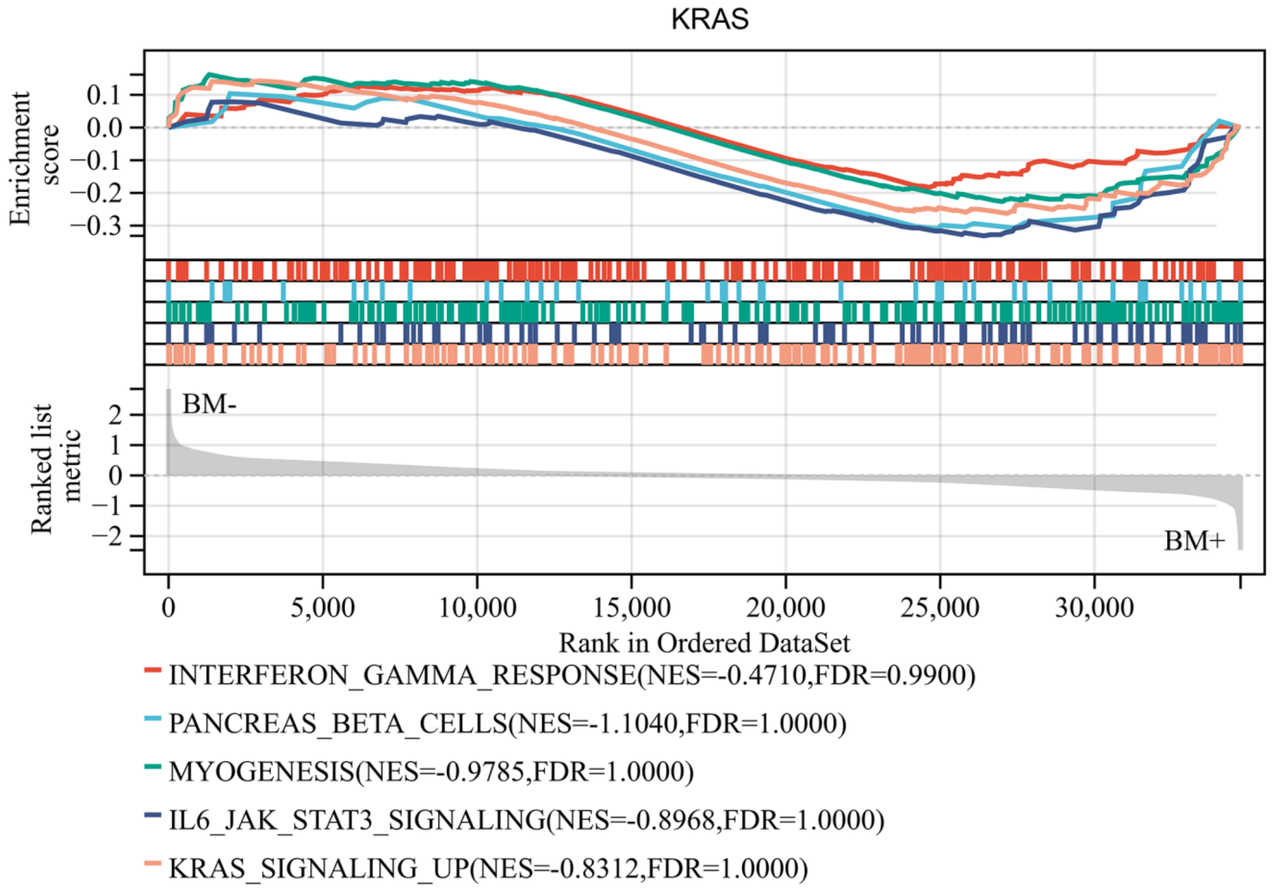

Supplementary Figure 2. GSEA analysis of KRAS showed that no significant gene sets were enriched based on GSE175601. Abbreviation: GSEA: Gene set enrichment analysis.

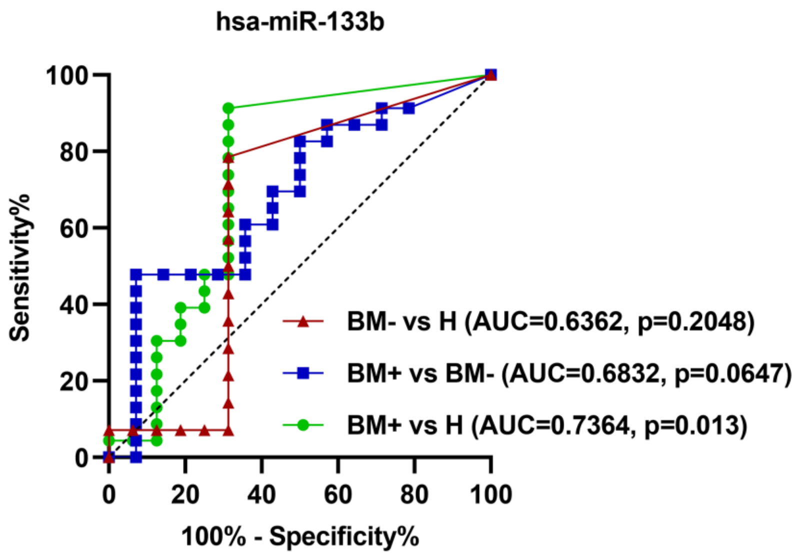

Supplementary Figure 3. ROC curves analysis shows that hsa-miR-133b could only significantly distinguish the BM+ group from healthy controls (H). ROC curve analysis of hsa-miR-133b. The ROC curves to discriminate the BM- group from H in the validation set are marked by red lines; the ROC curves to differentiate the BM+ group from the BM- group in the validation set are marked by blue lines; the ROC curves to differentiate the BM+ group from H in the validation set are marked by green lines. Abbreviations: ROC: receiver operator characteristic curve; BM+: patients with bone metastasis; BM-: patients without bone metastasis.
