# Supplementary material for: Exosomal hsa-miR-151a-3p and hsa-miR-877-5p are potential novel biomarkers for predicting bone metastasis in lung cancer
Source: Aging (Albany NY). 2023 Dec 18;15(24):14864–88. doi: 10.18632/aging.205314 (PMC10781484; doi:10.18632/aging.205314)
Supplement: Supplementary Tables [file aging-15-205314-s002.pdf]

SUPPLEMENTARY TABLES

Supplementary Table 1. The small RNA sequencing results of the top 10 differentially expressed miRNAs.

miRNA ID	Exo-A549-expression	Exo-95 D-expression	log2Ratio (Exo-95D/Exo-A549)	*P*-value	Regulation
hsa-miR-146b-5p	0.1	739.7681	12.85286	6.01E-45	Up
hsa-miR-376c-3p	0.1	616.4734	12.58982	1.37E-37	Up
hsa-miR-3180	0.1	487.014	12.24975	7.33E-30	Up
hsa-miR-3180-3p	0.1	487.014	12.24975	7.33E-30	Up
hsa-miR-495-3p	0.1	314.4014	11.61839	1.47E-19	Up
hsa-miR-154-5p	0.1	295.9072	11.53093	1.87E-18	Up
hsa-miR-654-3p	0.1	271.2483	11.4054	5.54E-17	Up
hsa-miR-200a-3p	0.1	265.0836	11.37223	1.29E-16	Up
hsa-miR-381-3p	0.1	240.4246	11.23137	3.83E-15	Up
hsa-miR-302d-3p	221.952	0.1	−11.116	2.46E-12	Down

Supplementary Table 2. 101 predicted target genes of the 29 candidate exosomal miRNAs.

Gene symbol	Gene symbol	Gene symbol	Gene symbol
ANKRD29	CUL5	PCDHAC1	SCARB1
ANKRD29	SOS2	BMF	DIO1
POGK	AP4E1	BRWD1	DPYSL2
SLC25A22	AGO1	BCL2L11	PPARGC1A
E2F5	PHACTR2	ARID1A	TMEM170A
SOX5	KLF6	POGZ	SDHD
PTBP2	BMP3	UBN2	SEMA6D
TAGLN2	FXR1	ZNF652	MMGT1
CNN2	DDX3X	FBXO28	STT3B
SP1	CCNL1	PANK3	RNF38
MCL1	ZNF544	ERBB4	SOCS6
FOXL2	RAB21	MIDN	FUT4
CPNE3	ETS1	ESR1	UQCRFS1
SUPT16H	PODXL	KIT	TMEM64
KPNA6	HIF1A	STMN1	TEX261
FOXC1	TSPAN3	FMR1	N4BP1
ARL6IP1	PAK4	ZFYVE16	JPH1
DDX6	RALGPS2	STOX2	TGFBR3
C1GALT1	PAN3	TLE3	PCMT1
SLC7A11	GID4	HIPK2	HCFC2
ZDHHC17	SATB1	PHACTR4	MAP3K2
LEPROTL1	NFIA	PPP2R2A	SH3BGRL3
MTMR10	TRPS1	DCAF7	KRAS
RCC2	MAPK10	GNAI3	
OSBPL11	NDFIP1	BBC3	
CEBPG	PCDHA3	FOXO1	

Supplementary Table 3. The top 5 enriched GO terms and KEGG pathways of 101 predicted target genes of the 29 candidate exosomal miRNAs.

Category	Term ID	Term description	Count	*P*-value
BP	GO:0045893	positive regulation of transcription, DNA-templated	15	1.21E-05
BP	GO: 0045944	positive regulation of transcription from RNA polymerase II promoter	18	8.36E-05
BP	GO: 0006357	regulation of transcription from RNA polymerase II promoter	21	2.72E-04
BP	GO:0051000	positive regulation of nitric-oxide synthase activity	4	2.87E-04
BP	GO:0001755	neural crest cell migration	4	0.00166
CC	GO: 0000785	chromatin	17	4.52E-05
CC	GO: 0005654	nucleoplasm	37	4.86E-05
CC	GO: 0005829	cytosol	43	5.23E-04
CC	GO:0005634	nucleus	45	7.39E-04
CC	GO:0000791	euchromatin	4	0.00353
MF	GO:0008134	transcription factor binding	10	1.12E-06
MF	GO:0043565	sequence-specific DNA binding	11	6.17E-06
MF	GO:0001228	transcriptional activator activity, RNA polymerase II transcription regulatory region sequence-specific binding	12	2.64E-05
MF	GO:0005515	protein binding	80	2.46E-04
MF	GO:0033592	RNA strand annealing activity	3	2.50E-04
KEGG	hsa05200	Pathways in cancer	12	1.63E-04
KEGG	hsa05211	Renal cell carcinoma	5	6.36E-04
KEGG	hsa04917	Prolactin signaling pathway	5	6.72E-04
KEGG	hsa04012	ErbB signaling pathway	5	0.00139
KEGG	hsa05210	Colorectal cancer	5	0.00145
